# Comparison of Bacteriology Between Geriatric and Adult Rhinosinusitis with and Without NPs

**DOI:** 10.3390/diagnostics15233035

**Published:** 2025-11-28

**Authors:** Meng-Chun Lin, Yi-Ching Chen, Rong-San Jiang

**Affiliations:** 1 Department of Otolaryngology, Tungs’ Taichung Metro Harbor Hospital, Taichung 435403, Taiwan; mons0079@gmail.com; 2 Department of Medical Research, Tungs’ Taichung Metro Harbor Hospital, Taichung 435403, Taiwan; cf0817@gmail.com; 3 Department of Otolaryngology, Taichung Veterans General Hospital, Taichung 407219, Taiwan; 4 Department of Medical Research, Taichung Veterans General Hospital, Taichung 407219, Taiwan; 5 School of Medicine, Chung Shan Medical University, Taichung 402306, Taiwan

**Keywords:** adult, bacteriology, chronic rhinosinusitis, geriatric, nasal polyp

## Abstract

**Background**: In this study, we attempted to compare the bacteriology of chronic rhinosisusitis (CRS) with and without nasal polyps between geriatric and adult patients. **Methods**: This retrospective cross-sectional study included 751 patients with CRS who underwent bilateral primary functional endoscopic sinus surgery. Before surgery, swab samples were collected from the middle meatus for bacterial cultures using cotton-tipped sticks. Subjects were divided into adult (20 to 64 years, *n* = 683) and elderly (65 years, *n* = 68) groups. The results of the bacteria culture were analyzed according to age group and the presence of nasal polyps. **Results**: The bacterial culture rate was higher in geriatric patients (55.9%) than in adults (44.9%), but the difference was not statistically significant. However, geriatric patients showed a higher bacterial culture rate (57.6%) than adult patients (29.6%) without nasal polyps. This difference was statistically significant. **Conclusions**: Geriatric patients with CRS exhibited higher bacterial culture rates, particularly on the non-polyp side. These findings suggest a possible age-related susceptibility to microbial colonization, underscoring the need for age-specific infection management strategies.

## 1. Introduction

The elderly population is increasing in the world. The 2014 US census reported that elderly citizens comprised 20% of the population in the United States [1]. In Taiwan, people aged > 65 years of age will reach 30% in 2039, according to a report by the National Development Council. Moreover, chronic rhinosinusitis (CRS) represents one of the most prevalent chronic conditions [2]. It is estimated that the condition affects between 14–16% of Americans and 10.9% of Europeans [3]. CRS is reportedly the sixth most-prevalent disease impacting the elderly population [4].

The etiology of CRS is attributed to multiple factors [5]. These include bacterial and fungal infections or colonization, allergies, anatomical abnormalities, and a genetic predisposition [2,6,7]. CRS has been classified based on the existence or non-existence of nasal polyps as CRS with nasal polyps (CRSwNP) or CRS without nasal polyps (CRSsNP) [8]. It is believed that bacteria play a significant role in the development of CRS [9,10], and the formation of biofilms, the secretion of superantigens, and the disruption of the nasal microbiota’s balance may all contribute to CRS [11,12]. Comparisons between CRSwNP and CRSsNP have been made regarding their bacteriology. Niederfuhr et al. [13] and Liu et al. [14] found no difference in microbiological features between CRSwNP and CRSsNP, whereas Stern et al. [15] discovered that CRSwNP is linked to a greater presence of Gram-negative bacteria compared to CRSsNP.

When studying CRS in older adults, it is essential to take into account the impact of natural aging on the immune system in order to understand the causes of geriatric CRS [16]. Factors that increase the risk of geriatric CRS include changes to the nasal and paranasal mucosa, including atrophy of the nasal mucosa, reduced mucus production, and impaired mucociliary clearance. The fibro-fatty tissues supporting the nose can degenerate, leading to potential loss of support for nasal structures and associated nasal blockage [17]. People over a certain age with reduced immunity to infections and a higher number of co-existing health conditions typically experience a more severe clinical manifestation of CRS [16]. Despite a lack of investigation, age-related variations in nasal colonization are particularly unclear in elderly patients with or without nasal polyps. We hypothesized that elderly patients with chronic rhinosinusitis would have higher nasal bacterial culture positivity rates compared to adults due to decreased mucosal immunity and microbiome alteration with age. In this investigation, we attempted to explore the bacteriology of geriatric chronic rhinosinusitis (CRS), comparing geriatric cases with and without nasal polyps to those of adults in order to determine the role of bacteria in the development of CRS in the elderly population.

## 2. Materials and Methods

### 2.1. Patients

Study participants were patients with CRS who had failed adequate medical treatment and subsequently underwent bilateral primary functional endoscopic sinus surgery between September 2005 and February 2025. A diagnosis of chronic rhinosinusitis (CRS) was made using patient history, nasal endoscopy, and a sinus CT scan [18]. Typically, medical treatment required management with intranasal steroids and nasal saline irrigation for a period of at least 3 months. Certain patients may also receive low-dose macrolides [19]. We excluded patients with a history of immunodeficiency or who had undergone sinus surgery, as well as those who had received antibiotic treatment within one week prior to FESS. Individuals diagnosed with a pathological condition of fungal sinusitis or sinonasal tumor were also eliminated from the study. Patients who were eligible for CRS treatment were categorized into two groups—adults, who were 65 years of age or younger, and the geriatric group, whose ages exceeded 65 years—which were classified as chronic rhinosinusitis with nasal polyps (CRSwNP) or chronic rhinosinusitis without nasal polyps (CRSsNP) based on the presence of nasal polyps. The Institutional Review Board of Taichung Veterans General Hospital gave approval (approval No. CE22515A) for this study, which used medical records from the Department of Otorhinolaryngology.

### 2.2. Evaluation of the Severity of Rhinosinusitis

The severity of rhinosinusitis was assessed before surgery via endoscopic examination and CT scan. The Lund–Kennedy staging system rated endoscopic appearances on a scale of 0 to 2 points [20]. Three scoring domains included the presence of polyps (0 signifying no polyps; 1 representing polyps confined to the middle meatus; 2 indicating polyps beyond the middle meatus), discharge (0 indicating no discharge; 1 denoting clear, thin discharge; 2 indicating thick, purulent discharge), and edema. The total endoscopic score for each nasal cavity was the sum score of 3 domains in the nasal cavity on the same side (range 0–6). Patients were categorized into two groups based on the presence of nasal polyps visible during endoscopy: those with nasal polyps and those without. The sinus CT was evaluated using the Lund–Mackay scoring system [21]. The five sinus groups, namely the maxillary, anterior ethmoid, posterior ethmoid, sphenoid, and frontal sinuses, were each graded on a scale of 0 to 2 (0: clear sinus; 1: partial opacification; 2: total opacification). The ostiomeatal complex was rated as either 0 (not blocked) or 2 (obstructed). The total CT score was the sum score of 6 domains in the nasal cavity on one side (range 0–12).

### 2.3. Nasal Bacterial Culture

Swab samples were collected the day prior to surgery from both middle meati using cotton-tipped sticks. The sticks were then inserted into Thanswab tubes containing 5 mL of Amies charcoal medium, which is used for the culture of both aerobes and anaerobes, before the tubes were sent to the clinical microbiology laboratory [22]. Upon arrival at the laboratory, the sticks in the Thanswab tubes were swabbed onto 5% sheep blood agar, eosin methylene blue agar, and chocolate agar plates. The plates were then incubated in a CO_2_ (5%) incubator at 350 °C for 2 and 4 days. An anaerobic Brucella blood agar was inoculated for anaerobes and then incubated in a Form anaerobic system for 2 and 4 days. The samples were then placed in a thioglycolate broth tube for the enrichment of anaerobes, and incubated at 35 °C for 2 days. In the laboratory, all isolates were examined and identified, including checking for anaerobes and both aerobic and facultative bacteria. A culture result was deemed positive if any aerobic or facultative bacteria or anaerobes grew, and it was considered negative if neither aerobic nor facultative bacteria nor anaerobes grew. The bacterial culture rate was determined by dividing the number of samples with positive cultures by the total number of samples.

### 2.4. Statistical Analyses

Patients were classified into four subgroups according to age and the presence of nasal polyps: adult CRSsNP, adult CRSwNP, geriatric CRSsNP, and geriatric CRSwNP. Comparisons were performed between specific pairs of these subgroups: (1) adult CRSwNP vs. adult CRSsNP, (2) geriatric CRSwNP vs. geriatric CRSsNP, (3) adult CRSwNP vs. geriatric CRSwNP, and (4) adult CRSsNP vs. geriatric CRSsNP.

All statistical analyses were performed using SPSS version 22 (IBM Corp., Armonk, NY, USA). Continuous variables were expressed as mean ± standard deviation (SD), and categorical variables as numbers and percentages. Continuous variables were analyzed using the independent *t*-test or Mann–Whitney U test, depending on data distribution, and categorical variables were compared using the Pearson chi-square test or Fisher’s exact test, as appropriate. Additional within-patient comparisons between nostrils with and without nasal polyps were performed using the same statistical approach. A two-tailed *p* value < 0.05 was considered statistically significant.

## 3. Results

### 3.1. Patients

Seven hundred and fifty-one CRS patients were enrolled in the study. Among them, 68 were patients with geriatric CRS, and the other 683 were those with adult CRS. Among the geriatric patients, 35 (51.5%) were CRSwNP, and 349 (51.1%) adults were CRSwNP. There was no significant difference in the prevalence of nasal polyps between patients with geriatric CRS and those with adult CRS (*p* = 1). Nasal polyps were present in 59 (43.4%) nostrils of patients with geriatric CRS and 486 (35.6%) nostrils of patients with adult CRS. The prevalence of nasal polyps in nostrils was not significantly different between patients with geriatric CRS and those with adult CRS (*p* = 0.087). The bacteriologies of geriatric CRS with and without nasal polyps and CRSsNP and adult CRS with and without nasal polyps are shown in Table 1.

### 3.2. Comparison Between Patients with Geriatric CRS and Patients with Adult CRS

Table 2 presents a comparison between geriatric patients with CRS and adult patients with CRS. Of the 68 geriatric patients with CRS, 43 (63.2%) were male and 25 (36.8%) were female. The ages spanned 65 to 84 years, with a mean of 70.34 ± 5.28 years. Of the 683 adults with CRS, 435 were men (63.7%) and 248 were women (36.3%). The ages spanned from 20 to 64 years with a mean of 43.84 ± 11.81 years. Of the geriatric patients, 35 (51.5%) were classified as having CRSwNP, and 349 (51.1%) of the adults also had CRSwNP. The scores for the bilateral nostrils in patients with geriatric chronic rhinosinusitis ranged from 2 to 10, and from 1 to 12 in those with adult CRS. The total CT score for bilateral nostrils fluctuated between 0 and 24 in patients with geriatric CRS and between 0 and 24 in those with adult CRS. In patients with geriatric CRS, the rate of bacterial culture was 55.9%, whereas in those with adult CRS, it was 44.9%. No significant differences were observed in the endoscopic score, CT score, and bacterial culture rate between patients with geriatric chronic rhinosinusitis (CRS) and those with adult CRS.

### 3.3. Comparison Between Patients with Geriatric CRSwNP and Patients with Geriatric CRSsNP

Table 3 shows the comparison between patients with geriatric CRSwNP and patients with geriatric CRSsNP. Among the 68 patients with geriatric CRS, 35 (51.5%) had CRSwNP and 33 (48.5%) had CRSsNP. Among the geriatric patients with CRSwNP, 27 (77.1%) were men and 8 (22.9%) were women, with ages spanning from 65 to 84 years and averaging 70.8 ± 5.34 years. Of the 33 patients with geriatric CRSsNP, 16 (48.5%) were male and 17 (51.5%) were female, with ages spanning 65 to 84 years and averaging 69.85 ± 5.26 years. The total endoscopic score of the bilateral nostrils ranged from 3 to 10 in patients with geriatric CRSwNP and from 2 to 7 in those with geriatric CRSsNP. The total CT score of the bilateral nostrils ranged from 4 to 24 in patients with geriatric CRSwNP and from 0 to 18 in those with geriatric CRSsNP. The rate of bacterial culture was 54.3% in patients with geriatric CRSwNP and 57.6% in those with geriatric CRSsNP. Endoscopic and CT scores were significantly higher in patients with geriatric CRSwNP than in those with geriatric CRSsNP, but there was no significant difference in the bacterial culture rate between patients with geriatric CRSwNP and those with geriatric CRSsNP.

### 3.4. Comparison Between Patients with Adult CRSwNP and Patients with Adult CRSsNP

Table 4 shows the comparison between patients with adult CRSwNP and patients with adult CRSsNP. Among the 683 adults with CRS, 349 (51.1%) had CRSwNP and 334 (48.9%) had CRSsNP. There were 235 (67.3%) men and 114 (32.7%) women in adult patients with CRSwNP, and the age ranged from 20 to 64 years with a mean of 44.02 ± 11.83 years. Of the 334 adults with CRSsNP, 200, or 59.9%, were men and 134, or 40.1%, were women, with a range of ages spanning from 20 to 64 years and a mean age of 43.63 ± 11.80 years. The total endoscopic score of the bilateral nostrils ranged from 1 to 12 in patients with adult CRSwNP and from 2 to 9 in those with adult CRSsNP. The total CT score of the bilateral nostrils ranged from 0 to 24 in patients with adult CRSwNP and from 0 to 22 in those with adult CRSsNP. The bacterial culture rate was 59.6% in patients with adult CRSwNP and 29.6% in those with adult CRSsNP. Endoscopic score, CT score, and bacterial culture rate were significantly higher in patients with adult CRSwNP than in those with adult CRSsNP.

### 3.5. Comparison Between Patients with Geriatric CRSwNP and Patients with Adult CRSwNP

Table 5 shows the comparison between patients with geriatric CRSwNP and patients with adult CRSwNP. There were 35 patients with geriatric CRSwNP and 349 patients with adult CRSwNP. Among the 35 patients with geriatric CRSwNP, there were 27 (77.1%) males and 8 (22.9%) females, and the age ranged from 65 to 84 years old with a mean of 70.8 ± 5.34 years. Among the 349 patents with adult CRSwNP, there were 235 (67.3%) men and 114 (32.7%) women, and the age ranged from 20 to 64 years with a mean of 44.02 ± 11.83 years. The total endoscopic score of the bilateral nostrils ranged from 3 to 10 in patients with geriatric CRSwNP and from 1 to 12 in patients with adult CRSwNP. The total CT score of the bilateral nostrils ranged from 4 to 24 in patients with geriatric CRSwNP and from 0 to 24 in patients with adult CRSwNP. The bacterial culture rate was 54.3% in patients with geriatric CRSwNP and 59.6% in those with adult CRSwNP. Endoscopic and CT scores were significantly higher in patients with geriatric CRSwNP than in those with adult CRSwNP, but there were no significant differences in the bacterial culture rate between patients with geriatric CRSwNP and those with adult CRSwNP.

### 3.6. Comparison Between Patients with Geriatric CRSsNP and Patients with Adult CRSsNP

Table 6 shows the comparison between patients with geriatric CRSsNP and patients with adult CRSsNP. There were 33 patients with geriatric CRSsNP and 334 patients with adult CRSsNP. Among the 33 patients with geriatric CRSsNP, there were 16 (48.5%) males and 17 (51.5%) females, and the age ranged from 65 to 84 years old with a mean of 69.85 ± 5.26 years. Among the 334 patents with adult CRSsNP, there were 200 (59.9%) males and 134 (40.1%) females, and the age ranged from 20 to 64 years old with a mean of 43.63 ± 11.80 years. The total endoscopic score of the bilateral nostrils ranged from 2 to 7 in patients with geriatric CRSsNP and from 2 to 9 in patients with adult CRSsNP. The total CT score of the bilateral nostrils ranged from 0 to 18 in patients with geriatric CRSsNP and from 0 to 22 in patients with adult CRSsNP. The bacterial culture rate was 57.6% in patients with geriatric CRSsNP and 29.6% in those with adult CRSsNP. There were no significant differences in endoscopic and CT scores between patients with geriatric CRSsNP and those with adult CRSsNP, but the bacterial culture rate was significantly higher in patients with geriatric CRSsNP than in those with adult CRSsNP.

### 3.7. Comparison Between Nostrils with Nasal Polyps and Nostrils Without Nasal Polyps in Patients with Geriatric CRS

Table 7 shows the comparison between nostrils with nasal polyps and nostrils without nasal polyps in patients with geriatric CRS. Among the 68 patients with geriatric CRS, nasal polyps were present in 59 (43.4%) of the 136 nostrils. The endoscopic score of each nostril ranged from 2 to 5 in nostrils with nasal polyps and from 0 to 4 in nostrils without nasal polyps. The CT score of each nostril ranged from 1 to 12 in nostrils with nasal polyps and from 0 to 10 in nostrils without nasal polyps. The bacterial culture rate was 44.1% in nostrils with nasal polyps and 49.4% in nostrils without nasal polyps. Endoscopic and CT scores were significantly higher in nostrils with nasal polyps than in those without nasal polyps, however the rate of positive bacterial cultures did not significantly between the two groups.

### 3.8. Comparison Between Nostrils with Nasal Polyps and Nostrils Without Nasal Polyps in Patients with Adult CRS

Table 8 shows the comparison between nostrils with nasal polyps and nostrils without nasal polyps in patients with adult CRS. Among the 683 patients with adult CRS, nasal polyps were present in 486 (35.6%) of the 1366 nostrils. The endoscopic score of each nostril ranged from 1 to 6 in nostrils with nasal polyps and from 0 to 5 in nostrils without nasal polyps. The CT score of each nostril ranged from 0 to 12 in nostrils with nasal polyps and from 0 to 12 in nostrils without nasal polyps. The bacterial culture rate was 53.9% in nostrils with nasal polyps and 23.9% in nostrils without nasal polyps. The endoscopic score, CT score, and bacterial culture rate were significantly higher in nostrils with nasal polyps than in those without nasal polyps.

### 3.9. Comparison of Nostrils with Nasal Polyps Between Patients with Geriatric CRS and Patients with Adult CRS

Table 9 shows the comparison of nostrils with nasal polyps between patients with geriatric CRS and patients with adult CRS. Among the 59 nostrils with nasal polyps in patients with geriatric CRS, the endoscopic score of each nostril ranged from 2 to 5 and the CT score of each nostril ranged from 1 to 12. The bacterial culture rate was 44.1%. Among the 486 nostrils with nasal polyps in patients with adult CRS, the endoscopic score of each nostril ranged from 1 to 6 and the CT score of each nostril ranged from 0 to 12. The bacterial culture rate was 53.9%. Endoscopic and CT scores were significantly higher in nostrils with nasal polyps in patients with geriatric CRS than in nostrils with nasal polyps in those with adult CRS, but there was no significant difference in the rate of bacterial culture of the nostrils with nasal polyps between patients with geriatric CRS and patients with adult CRS.

### 3.10. Comparison of Nostrils Without Nasal Polyps Between Patients with Geriatric CRS and Patients with Adult CRS

Table 10 shows the comparison of nostrils without nasal polyps between patients with geriatric CRS and patients with adult CRS. Among the 77 nostrils without nasal polyps in patients with geriatric CRS, the endoscopic score of each nostril ranged from 0 to 4 and the CT score of each nostril ranged from 0 to 10. The bacterial culture rate was 49.4%. Among the 880 nostrils without nasal polyps in patients with adult CRS, the endoscopic score of each nostril ranged from 0 to 5 and the CT score of each nostril ranged from 0 to 12. The bacterial culture rate was 23.9%. There were no significant differences in endoscopic and CT scores in nostrils without nasal polyps between patients with geriatric CRS and patients with adult CRS, but the bacterial culture rate of nostrils without nasal polyps was significantly higher in patients with geriatric CRS than in those with adult CRS.

## 4. Discussion

Several factors contribute to the elderly being predisposed to CRS [23]. Changes in the nasal and paranasal mucosa with age involve: (1) a rise in volume coupled with a drop in elasticity of the nasal mucosa; (2) a diminished or non-existent nasal cycle, partly resulting from a decrease in ciliary effectiveness; (3) atrophy of the supporting tissues of the nose and a potential loss of support for nasal structures, leading to increased nasal obstruction; (4) a higher frequency of rhinorrhea with more mucus due to heightened glandular activity and more viscous secretions; and (5) excessive mucus crusting [24,25].Therefore, it is assumed that geriatric CRS has different pathophysiology and clinical presentations from adult CRS [26]. Nasal polyps were reported to be more prevalent in patients with geriatric CRS compared to those with adult CRS and that CT scan scores of patients with geriatric CRS were significantly worse than those with adult CRS [27,28,29]. In contrast, our results showed nasal polyps were not more prevalent in patients with geriatric CRS than in those with adult CRS, and CT scan scores of patients with geriatric CRS were not significantly worse than those with adult CRS. However, the CT and endoscopic scores of our geriatric patients with CRSwNP were significantly worse than those of our adult patients with adult CRSwNP.

Because innate and adaptive immune responses may be altered in geriatric people, the susceptibility to infection and bacterial colonization increases [30]. Beyond this general statement, age-related immunologic remodeling also affects hypersensitivity mechanisms that participate in CRS pathophysiology. Aging is associated with progressive impairment of epithelial barrier integrity, reduced antigen presentation, and dysregulated T helper cell polarization. These changes can modify the balance among Th1, Th2, and Th17 pathways, resulting in altered hypersensitivity profiles. In particular, age-related reductions in IgE synthesis and eosinophil recruitment may attenuate classical type I hypersensitivity, whereas diminished regulatory T-cell activity and persistent low-grade inflammation (“inflammaging”) can enhance non-IgE-mediated chronic inflammation of the sinonasal mucosa.

In elderly patients with CRSsNP, the higher bacterial culture rate observed in our study may reflect a shift from an eosinophilic-dominant to a neutrophilic-dominant inflammatory pattern, driven by immunosenescence and mucociliary dysfunction. This shift could favor persistent bacterial colonization and biofilm formation rather than eosinophil-mediated mucosal inflammation typically seen in adults. Clinically, this suggests that infection control and microbiome-targeted therapies—such as prolonged low-dose macrolides, topical antiseptics, or mucociliary clearance enhancement—might be more beneficial in geriatric CRSsNP than solely anti-inflammatory regimens. These findings highlight that age-related immune remodeling not only increases infection susceptibility but also alters the inflammatory landscape of CRS, underscoring the need for age-specific management strategies.

Analysis of the bacterial culture results detailed in Table 1 revealed distinct patterns of colonization across the four patient subgroups. Specifically, patients with adult CRSwNP (59.6%) demonstrated a significantly higher rate of positive cultures compared to those with adult CRSsNP (29.6%) (*p* < 0.05), establishing an association between nasal polyps and increased bacterial colonization in the adult population; conversely, no statistically significant difference in culture positivity was observed between geriatric CRSwNP (54.3%) and geriatric CRSsNP (57.6%) patients. Crucially, geriatric CRSsNP patients exhibited a significantly higher rate of positive bacterial cultures than their adult CRSsNP counterparts (*p* < 0.01), which represents a key finding, though no difference was detected when comparing the two CRSwNP groups. This indicated that bacteria played a different role between geriatric CRS and adult CRS, especially in CRSsNP. Therefore, the treatment of geriatric patients with CRS may require different or additional therapeutic approaches [4,26].

*Proteus* spp. and *Pseudomonas aeruginosa* were reported to be more common in patients with geriatric CRS than those with adult CRS [16]. However, the occurrence of bacteria was not significantly different between patients with geriatric CRSwNP and those with geriatric CRSsNP, and between patients with adult CRSwNP and those with adult CRSsNP. Our results also showed that the occurrence of bacteria was not significantly different between patients with geriatric CRSwNP and those with geriatric CRSsNP, and between patients with adult CRSwNP and those with adult CRSsNP. In another study, less occurrence of Actinobacteria but greater abundance of Fusobacteria and Staphylococcus aureus were found in patients with geriatric CRSwNP than those with adult CRSwNP [30]. Conversely, our results did not show any difference in the occurrence of bacteria between patients with geriatric CRSwNP and those with adult CRSwNP.

When CRS was phenotypically classified into CRSwNP and CRSsNP, in this study, the culture specimen was taken from each nostril. Therefore, it was assumed that the culture results might be different between nostrils with nasal polyps and those without nasal polyps. However, our results showed that the comparison between CRSwNP and CRSsNP was not different from the comparison between nostrils with nasal polyps and those without nasal polyps.

Beyond bacterial infection, allergic mechanisms may also contribute to the development of CRS, particularly in the elderly. While our initial discussion referred to “allergic rhinitis” as a possible comorbidity, this term may be too narrow to capture the full range of allergic and hypersensitivity disorders recognized today. “Allergic disease,” as currently defined, includes multiple phenotypes that extend beyond nasal mucosal inflammation alone. A representative example is Central Compartment Atopic Disease (CCAD)—a recently characterized subtype of CRS localized to the central nasal compartment and strongly associated with inhalant allergy [31]. Unlike typical allergic rhinitis, CCAD demonstrates localized sinus involvement and a distinct immunopathologic profile.

Furthermore, according to the recent EAACI Position Paper on the Nomenclature of Allergic Diseases and Hypersensitivity Reactions [32], allergic diseases should be defined primarily by their underlying immune mechanisms rather than by the clinical site of manifestation. This modern framework conceptualizes allergic diseases as a continuum of hypersensitivity conditions affecting multiple organ systems, including both upper and lower airways. Within this perspective, chronic rhinosinusitis—including atopic subtypes such as CCAD—can be regarded as part of the broader allergy–disease spectrum. Integrating this immunologic framework into the interpretation of geriatric CRS highlights that immune senescence not only impairs host defense against pathogens but also reshapes hypersensitivity responses, thereby influencing disease expression in older adults.

This study has several limitations. First, this is a retrospective study, and comorbidities related to allergic or hypersensitivity diseases—such as allergic rhinitis, asthma, and specific phenotypes like CCAD—were not systematically evaluated. According to the updated EAACI position paper [32], allergic diseases encompass a wide range of immune mechanisms beyond allergic rhinitis; our design did not allow for differentiation according to this modern classification. We acknowledge that these unmeasured factors may have influenced the clinical and microbiological patterns observed, particularly in elderly patients where immune aging could modulate both allergic and infectious inflammation. Second, only patients with CRS who received a bilateral primary functional endoscopic sinus for treatment were enrolled in the study; therefore, our results might not be generalized to all patients with CRS or specific types of CRS. Finally, we did not classify our patients with CRSwNP as eosinophilic CRSwNP and non-eosinophilic CRSwNP. Hirotsu et al. [33] investigated the differences in bacteria between eosinophilic and non-eosinophilic CRSwNP cases. They found no difference in bacteriology between patients with eosinophilic CRSwNP and those with non-eosinophilic CRSwNP. In contrast, Liu et al. [14] isolated significantly more Gram-negative aerobic and facultative anaerobic bacteria from patients with CRSwNP who had normal blood eosinophil counts than from those with elevated blood eosinophils. Future prospective studies incorporating allergy testing, eosinophilic biomarkers, and molecular microbiome analysis may clarify these relationships.

Although this study used conventional bacterial culture to evaluate microbial colonization, it is important to acknowledge the methodological limitations of culture-based techniques. Many bacterial species inhabiting the sinonasal mucosa are non-culturable or require specific growth conditions that are not replicated in standard laboratory media. Recent advances in molecular microbiology—such as next-generation sequencing (NGS) and 16S rRNA gene analysis—have enabled a more comprehensive characterization of the nasal microbiome and revealed significant differences in microbial diversity among CRS phenotypes and endotypes [34,35]. These molecular approaches have shown that alterations in microbial community structure, rather than the presence of a single pathogen, may play an important role in the pathogenesis of CRS.

Therefore, while our culture-based results provide insight into the presence of viable bacterial species and their relative frequency across age groups, they cannot fully capture the complexity of the sinonasal microbiota. Integrating traditional culture methods with DNA-based or sequencing technologies in future studies would provide a more accurate picture of microbial–host interactions in geriatric CRS and may clarify whether age-related immune changes influence microbiome composition.

## 5. Conclusions

Our study showed that geriatric patients with CRSsNP exhibited higher bacterial culture rates than adult patients with CRSsNP. It suggests an age-dependent difference in the pathophysiology of CRS. This may be related to immune senescence, reduced mucociliary clearance, and a possible shift toward neutrophilic inflammation. Therefore, treatment of geriatric CRS should not simply mirror that of younger adults but should rather incorporate individualized infection control and mucosal support strategies. Further studies integrating molecular microbiome analysis and immune profiling are warranted to clarify these mechanisms and develop tailored therapeutic approaches for elderly CRS patients.

## Figures and Tables

**Table 1:** Bacteriologies of geriatric CRS with and without nasal polyps and adult CRS with and without nasal polyps.

Group	Geriatric CRSwNP	Geriatric CRSsNP	Adult CRSwNP	Adult CRSsNP
	(59 *)	(77)	(486)	(880)
Species	Number of Isolates			
Aerobic and facultative bacteria				
Gram-positive				
*Staphylococcus aureus*	7	13	62	39
Coagulase-negatives *taphylococci*	4	6	60	45
*Staphylococcus* not *aureus*			1	2
*Staphylococcus lugdunensis*			1	
*Staphylococcus sciuri*			1	
*Streptococcus pneumoniae*	1	1	8	11
*Streptococcus viridans*			1	
Group B *streptococcus*			1	
Moraxellacatarrhalis		1	7	2
* Moraxella* spp.				1
*Corynebacterium* spp.	1	3	7	8
*Corynebacterium pseudodiphtheriticum*			2	2
*Enterococcus* spp.				2
Gram-positive *bacillus*	2			1
Gram-positive *coccus*	1			
Total Gram-positive bacteria	16	24	151	114
Gram-negative				
Haemophilus influenzae	4		14	12
* Citrobacter koseri*	1	3	22	18
* Citrobacter freundii*			3	
* Klebsiella pneumonia*	2		11	11
* Klebsiella oxytoca*	1	1	2	1
* Klebsiella ozaenae*				1
* Escherichia coli*	1	2	6	2
* Escherichia vulneris*			1	
* Enterobacter aerogenes*	1		8	12
* Enterobacter cloacae*	2		4	
* Pantoea agglomerans*			1	2
* Pseudomonas aeruginosa*		3	4	5
* Pseudomonas oryzihabitans*			1	
* Pseudomonas* spp.		1		1
* Shewanella algae*			4	
* Proteus vulgaris*			2	
* Proteus mirabilis*		1	1	1
*Bacillus* spp.			2	1
* Pasteurella multocida*		2		
Non-fermentative Gram-negative *Bacillus*			4	2
*Flavobacterium* spp.			1	
* Stenotrophomonas maltophilia*			1	
* Acinetobacter baumann*		2	1	2
* Serratia marcescens*				2
* Aeromonas hydrophila*				1
Total Gram-negative bacteria	12	15	93	75
Total aerobic and facultative bacteria	28	39	244	189
				
Anaerobic bacteria				
Gram-positive				
* Propionibacterium acnes*	2	3	25	20
* Propionibacterium granulosum*				2
*Propionibacterium* spp.			2	4
* * *Peptostreptococcus magnus*			6	3
* * *Peptostreptococcus micros*			3	1
* * *Peptostreptococcus anaerobius*			3	1
* * *Pavimonas micra*		1		3
* * *Clostridium sordellii*			1	
* * *Clostridium baratii*				1
* * *Clostridium perfringens*				1
* * *Finegoldia magna*			1	3
* Streptococcus constellatus*				1
* Streptococcus intermedius*				2
* Gemella morbillorum*				1
* Actinomyces odontolyticus*			1	1
* Slackia exigua*				2
Gram-negative				
* Fusobacterium nucleatum*		2		4
* Fusobacterium varium*			1	1
* Fusobacterium necrophorum*				1
* Fusobacterium* spp.			1	
* Prevotella intermedia*		1		1
* Prevotella oris*			1	
* Prevotella* spp.				1
* * *Veillonella atypica*			2	1
* * *Veillonella dispar*				1
* Veillonella* spp.			1	2
* Bacteroides thetaiotaomicron*			1	
* Porphyromonas gingivalis*				1
Total anaerobic bacteria	2	7	49	59
Total bacterial isolates	30	46	293	248

* number of specimens; CRS: chronic rhinosinusitis; CRSwNP: chronic rhinosinusitis with nasal polyps; CRSsNP: chronic rhinosinusitis without nasal polyps.

**Table 2:** Comparison between patients with geriatric CRS and patients with adult CRS.

	Geriatric CRS	Adult CRS	*p*-Value
Sex			1
Male	43 *	435	
Female	25	248	
Age (years old)	70.34 ± 5.28	43.84 ± 11.81	<0.001
Nasal polyps	35	349	1
Endoscopic score	6.10 ± 2.52	5.60 ± 2.21	0.143
CT score	11.15 ± 6.44	9.94 ± 5.98	0.096
Culture rate	38/68	307/683	0.110

*: number of patients.

**Table 3:** Comparison between patients with geriatric CRSwNP and patients with geriatric CRSsNP.

	Geriatric CRSwNP	Geriatric CRSsNP	*p*-Value
Sex			0.028
Male	27 *	16	
Female	8	17	
Age (years old)	70.80 ± 5.34	69.85 ± 5.26	0.302
Endoscopic score	7.89 ± 2.00	4.21 ± 1.36	<0.001
CT score	14.91 ± 4.73	7.15 ± 5.56	<0.001
Culture rate	19/35	19/33	0.110

*: number of patients; CRSwNP: chronic rhinosinusitis with nasal polyps; CRSsNP: chronic rhinosinusitis without nasal polyps.

**Table 4:** Comparison between patients with adult CRSwNP and patients with adult CRSsNP.

	Adult CRSwNP	Adult CRSsNP	*p*-Value
Sex			0.052
Male	235 *	200	
Female	114	134	
Age (years old)	44.02 ± 11.83	43.63 ± 11.80	0.617
Endoscopic score	6.70 ± 2.17	4.45 ± 1.59	<0.001
CT score	11.85 ± 5.84	7.94 ± 5.45	<0.001
Culture rate	208/349	99/334	<0.001

*: number of patients; CRSwNP: chronic rhinosinusitis with nasal polyps; CRSsNP: chronic rhinosinusitis without nasal polyps.

**Table 5:** Comparison between patients with geriatric CRSwNP and patients with adult CRSwNP.

	Geriatric CRSwNP	Adult CRSwNP	*p*-Value
Sex			0.318
Male	27 *	235	
Female	8	114	
Age (years old)	70.8 ± 5.34	44.02 ± 11.83	<0.001
Endoscopic score	7.89 ± 2.00	6.70 ± 2.17	0.002
CT score	14.91 ± 4.73	11.85 ± 5.84	0.003
Culture rate	19/35	208/349	0.668

*: number of patients; CRSwNP: chronic rhinosinusitis with nasal polyps.

**Table 6:** Comparison between geriatric CRSsNP and adult CRSsNP.

	Geriatric CRSsNP	Adult CRSsNP	*p*-Value
Sex			0.279
Male	16 *	200	
Female	17	134	
Age (years old)	69.85 ± 5.26	43.63 ± 11.80	<0.001
Endoscopic score	4.21 ± 1.36	4.45 ± 1.59	0.479
CT score	7.15 ± 5.56	7.94 ± 5.45	0.548
Culture rate	19/33	99/334	0.002

*: number of patients; CRSsNP: chronic rhinosinusitis without nasal polyps.

**Table 7:** Comparison between nostrils with nasal polyps and nostrils without nasal polyps in patients with geriatric CRS.

	Nostrils with NP	Nostrils Without NP	*p*-Value
Endoscopic score	4.29 ± 0.87	2.10 ± 0.84	<0.001
CT score	7.76 ± 2.72	3.90 ± 2.85	<0.001
Culture rate	26/59	38/77	0.661

CRS: chronic rhinosinusitis; NP: nasal polyps.

**Table 8:** Comparison between nostrils with nasal polyps and nostrils without nasal polyps in patients with adult CRS.

	Nostrils with NP	Nostrils Without NP	*p*-Value
Endoscopic score	3.62 ± 1.18	2.35 ± 0.99	<0.001
CT score	6.38 ± 3.00	4.19 ± 3.05	<0.001
Culture rate	262/486	210/880	<0.001

CRS: chronic rhinosinusitis; NP: nasal polyps.

**Table 9:** Comparison of nostrils with nasal polyps between patients with geriatric CRS and patients with adult CRS.

	Geriatric CRS	Adult CRS	*p*-Value
Endoscopic score	4.29 ± 0.87	3.62 ± 1.18	<0.001
CT score	7.76 ± 2.72	6.38 ± 3.00	<0.001
Culture rate	26/59	262/486	0.196

CRS: chronic rhinosinusitis.

**Table 10:** Comparison of nostrils without nasal polyps between patients with geriatric CRS and patients with adult CRS.

	Geriatric CRS	Adult CRS	*p*-Value
Endoscopic score	2.10 ± 0.84	2.35 ± 0.99	0.087
CT score	3.90 ± 2.85	4.19 ± 3.05	0.511
Culture rate	38/77	210/880	<0.001

CRS: chronic rhinosinusitis.

## Data Availability

The data sets used and analyzed in this study are available from the corresponding author upon reasonable request.

## Abbreviations

The following abbreviations are used in this manuscript:

CRS	Chronic rhinosinusitis
CRSsNP	Chronic rhinosinusitis without nasal polyps
CRSwNP	Chronic rhinosinusitis with nasal polyps
CT	Computed tomography

## References

[1] Hsu D.W., Suh J.D. (2018). Rhinitis and sinusitis in the geriatric population. Otolaryngol. Clin. N. Am..

[2] Dżaman K., Zagor M., Stachowiak M., Bielińska B., Sarnowska E., Krzeski A., Kantor I. (2016). The correlation of TAS2R38 gene variants with higher risk for chronic rhinosinusitis in Polish patients. Pol. J. Otolaryngol..

[3] Gallo S., Grossi S., Montrasio G., Binelli G., Cinquetti R., Simmen D., Castelnuovo P., Campomenosi P. (2016). TAS2R38 taste receptor gene and chronic rhinosinusitis: New data from an Italian population. BMC Med. Genet..

[4] Helman S.N., Carlton D., Deutsch B., Choake R., Patel V., Govindaraj S., Iloreta A.M.C., Del Signore A. (2021). Geriatric Sinus Surgery: A review of demographic variables, surgical success and complications in elderly surgical patients. Allergy Rhinol..

[5] Purnell P.R., Addicks B.L., Zalzal H.G., Shapiro S., Wen S., Ramadan H.H., Setola V., Siderovski D.P. (2019). Single nucleotide polymorphisms in chemosensory pathway genes GNB3, TAS2R19, and TAS2R38 are associated with chronic rhinosinusitis. Int. Arch. Allergy Immunol..

[6] Yoo F., Suh J.D. (2017). What is the evidence for genetics in chronic rhinosinusitis?. Curr. Opin. Otolaryngol. Head Neck Surg..

[7] Jeruzal-Świątecka J., Fendler W., Pietruszewska W. (2020). Clinical role of extraoral bitter taste receptors. Int. J. Mol. Sci..

[8] Cho S.H., Hamilos D.L., Han D.H., Laidlaw T.M. (2020). Phenotypes of chronic rhinosinusitis. J. Allergy ClinImmunol. Pract..

[9] Wagner Mackenzie B., Baker J., Douglas R.G., Taylor M.W., Biswas K. (2019). Detection and quantification of Staphylococcus in chronic rhinosinusitis. Int. Forum Allergy Rhinol..

[10] Chalermwatanachai T., Zhang N., Holtappels G., Bachert C. (2015). Association of mucosal organisms with patterns of inflammation in chronic rhinosinusitis. PLoS ONE.

[11] Kim D.K., Wi Y.C., Shin S.J., Kim K.R., Kim D.W., Cho S.H. (2019). Diverse phenotypes and endotypes of fungus balls caused by mixed bacterial colonization in chronic rhinosinusitis. Int. Forum Allergy Rhinol..

[12] Tai J., Han M.S., Kwak J., Kim T.H. (2021). Association between microbiota and nasal mucosal diseases in terms of immunity. Int. J. Mol. Sci..

[13] Niederfuhr A., Kirsche H., Riechelmann H., Wellinghausen N. (2009). The bacteriology of chronic rhinosinusitis with and without nasal polyps. Arch. Otolaryngol. Head Neck Surg..

[14] Liu Q., Lu X., Bo M., Qing H., Wang X., Zhang L. (2014). The microbiology of chronic rhinosinusitis with and without nasal polyps. Acta Oto-Laryngol..

[15] Stern S., Hadar T., Nachalon Y., Ben-Zvi H., Soudry E., Yaniv E. (2016). Bacteriology of the middle meatus in chronic rhinosinusitis with and without polyposis. ORL J. Otorhinolaryngol. Relat. Spec..

[16] Leszczyńska J., Stryjewska-Makuch G., Ścierski W., Lisowska G. (2020). Bacterial flora of the nose and paranasal sinuses among patients over 65 years old with chronic rhinosinusitis who underwent endoscopic sinus surgery. Clin. Interv. Aging..

[17] Dhanuka A., Rout K., Jena D. (2020). Outcomes of endoscopic sinus surgery in geriatric patients: An institutional study. Indian J. Otolaryngol. Head Neck Surg..

[18] Benninger M.S., Ferguson B.J., Hadley J.A., Hamilos D.L., Jacobs M., Kennedy D.W., Lanza D.C., Marple B.F., Osguthorpe J.D., Stankiewicz J.A. (2003). Adult chronic rhinosinusitis: Definitions, diagnosis, epidemiology, and pathophysiology. Otolaryngol. Head Neck Surg..

[19] Fokkens W.J., Lund V.J., Hopkins C., Hellings P.W., Kern R., Reitsma S., Toppila-Salmi S., Bernal-Sprekelsen M., Mullol J., Alobid I. (2020). European Position Paper on Rhinosinusitis and Nasal Polyps 2020. Rhinology.

[20] Jiang R.S., Liang K.L. (2023). Comparison of bacteriology between eosinophilic and noneosinophilic chronic rhinosinusitis with nasal polyps. Otolaryngol Head Neck Surg..

[21] Tsuei L.H., Jiang R.S. (2025). The influence of allergic biomarkers in chronic rhinosinusitis patients who underwent functional endoscopic sinus surgery. OTO Open.

[22] Wang J.J., Chen C.Y., Liang K.L., Jiang R.S. (2020). Predictors of nasal bacterial culture rates in patients with chronic rhinosinusitis. Eur. J. ClinMicrobiol. Infect. Dis..

[23] Marioni G., Zanotti C., Brescia G. (2018). Chronic rhinosinusitis with nasal polyps in the elderly: Assessing current evidence. Allergy Asthma Proc..

[24] DelGaudio J.M., Panella N.J. (2016). Presbynasalis. Int. Forum Allergy Rhinol..

[25] Loftus P.A., Wise S.K., Nieto D., Panella N., Aiken A., DelGaudio J.M. (2016). Intranasal volume increases with age: Computed tomography volumetric analysis in adults. Laryngoscope.

[26] Hwang C.S., Lee H.S., Kim S.N., Kim J.H., Park D.J., Kim K.S. (2019). Prevalence and risk factors of chronic rhinosinusitis in the elderly population of Korea. Am. J. Rhinol. Allergy.

[27] Valdés C.J., Tewfik M.A. (2018). Rhinosinusitis and allergies in elderly patients. Clin. Geriatr. Med..

[28] Cho S.H., Kim D.W., Lee S.H., Kolliputi N., Hong S.J., Suh L., Norton J., Hulse K.E., Seshadri S., Conley D.B. (2015). Age-related increased prevalence of asthma and nasal polyps in chronic rhinosinusitis and its association with altered IL-6 trans-signaling. Am. J. Respir. Cell Mol. Biol..

[29] We J., Lee W.H., Tan K.L., Wee J.H., Rhee C.S., Lee C.H., Ahn S., Lee J.H., Kim J.W. (2015). Prevalence of nasal polyps and its risk factors: Korean National Health and Nutrition Examination Survey 2009–2011. Am. J. Rhinol. Allergy.

[30] Song W.J., Lee J.H., Won H.K., Bachert C. (2019). Chronic rhinosinusitis with nasal polyps in older adults: Clinical presentation, pathophysiology, and comorbidity. Curr. Allergy Asthma Rep..

[31] Davies C., Wu F., Huang E.Y., Takashima M., Rowan N.R., Ahmed O.G. (2023). Central Compartment Atopic Disease as a Pathophysiologically Distinct Subtype of Chronic Rhinosinusitis: A Scoping Review. Sinusitis.

[32] Jutel M., Agache I., Zemelka-Wiacek M., Akdis M., Chivato T., Del Giacco S., Gajdanowicz P., Gracia I.E., Klimek L., Lauerma A. (2023). Nomenclature of allergic diseases and hypersensitivity reactions: Adapted to modern needs: An EAACI position paper. Allergy.

[33] Hirotsu M., Kikuchi K., Kusunoki T., Kase K., Ono N., Ikeda K. (2011). Comparison of bacterial examinations between eosinophilic and neutrophilic chronic rhinosinusitis with nasal polyps. Acta Oto-Laryngol..

[34] Abreu N.A., Nagalingam N.A., Song Y., Roediger F.C., Pletcher S.D., Goldberg A.N., Lynch S.V. (2012). Sinus microbiome diversity depletion and Corynebacterium tuberculostearicum enrichment mediates rhinosinusitis. Sci. Transl. Med..

[35] Connell J.T., Bouras G., Yeo K., Fenix K., Cooksley C., Bassiouni A., Vreugde S., Wormald P.J., Psaltis A.J. (2024). Characterising the allergic fungal rhinosinusitis microenvironment using full-length 16S rRNA gene amplicon sequencing and fungal ITS sequencing. Allergy.

